# Proliferative Vasculopathy Associated With Antiphospholipid Antibodies in Patients With Neurological Symptoms

**DOI:** 10.3389/fmed.2022.913203

**Published:** 2022-06-20

**Authors:** Jina Yeo, Inpyeong Hwang, Chul-Ho Sohn, Eunyoung Emily Lee, Soon-Tae Lee, Eun Bong Lee, Jin Kyun Park

**Affiliations:** ^1^Division of Rheumatology, Department of Internal Medicine, Gil Medical Center, Gachon University College of Medicine, Incheon, South Korea; ^2^Division of Rheumatology, Department of Internal Medicine, Seoul National University Hospital and Seoul National University Hospital College of Medicine, Seoul, South Korea; ^3^Department of Radiology, Seoul National University Hospital, Seoul, South Korea; ^4^Department of Radiology, Seoul National University College of Medicine, and Institute of Radiation Medicine, Seoul National University Medical Research Center, Seoul, South Korea; ^5^Division of Rheumatology, Department of Internal Medicine, Korea Cancer Center Hospital, Korea Institute of Radiological and Medical Sciences, Seoul, South Korea; ^6^Department of Neurology, Seoul National University Hospital and College of Medicine, Seoul, South Korea

**Keywords:** antiphospholipid syndrome, CNS vasculopathy, magnetic resonance angiography, proliferative vasculopathy, stenosis and obstruction

## Abstract

**Background:**

Proliferative vasculopathy (PV) associated antiphospholipid syndrome (APS) in the central nervous system is a rare un(der)recognized form of extra-criteria manifestations of APS. This study investigated the angiographic characteristics of cerebral and cervical arteries in patients with PV associated with antiphospholipid antibodies (aPLs).

**Methods:**

Patients with aPLs, neurologic symptoms and diffuse luminal narrowing on brain or neck magnetic resonance angiography were selected from electronic medical records. Vascular wall and intraluminal pathology were examined by high-resolution vessel wall MR imaging (VW-MRI).

**Results:**

A total of 11 patients (six men and five women) with PV-aPL, of median (interquartile range) age 42 (34–61) years, were included. Median anticardiolipin antibodies IgG titer was 78.9 (28.2–134.0) units and anti-beta 2 glycoprotein I antibodies (aB2GPIs) IgG titer was 227.2 (0.0–1012.1) units. All patients had diffuse luminal narrowing in the carotid basilar and/or cerebral arteries, five in the internal carotid artery (ICA); two each in the middle cerebral artery (MCA) and vertebral artery; and one each in the basilar artery (BA) and posterior cerebral artery. On VW-MRI, four patients showed concentric thickening of the vascular walls of the ICA and/or MCA and two showed mild eccentric wall thickening of the ICA or BA. All patients received antithrombotic treatment. In two patients with extremely high aB2GPIs titer, diffuse narrowing progressed despite treatment with antithrombotic agents on follow-up imaging.

**Conclusions:**

This study suggests that PV-aPL might be a distinct extra-criteria manifestation of APS that can manifest as long-segmental diffuse stenosis of cerebral and cervical arteries. It should be considered in relatively young patients with neurologic symptoms and aPLs.

## Introduction

Antiphospholipid syndrome (APS) is an acquired autoantibody mediated thrombogenic condition, characterized by recurrent arterial or venous thromboembolism, and/or pregnancy morbidities, in the persistent presence of antiphospholipid antibodies (aPLs) (1). These aPLs bind to phospholipids of activated endothelial cells and activate the complement and coagulation cascades, leading to a hypercoagulable state with intraluminal thrombus formation (2).

Cerebrovascular accidents (CVA), including ischemic stroke and transient ischemic attack (TIA), are common central nervous system (CNS) manifestations of APS (3). High levels of anticardiolipin antibodies (aCLs) are associated with an increased risk of recurrent strokes (4). Interestingly, aPLs can activate endothelial cells (5) and induce the proliferation of vascular cells in the intima and media without intraluminal thrombus formation (6, 7). This non-thrombotic proliferative vasculopathy (PV) associated with aPL (PV-aPL) can lead to critical stenoses in the pulmonary, visceral and peripheral arteries (8). In contrast to an arterial thrombus of APS or an atherosclerotic plaque, which can induce an abrupt, short-segmental stenosis or occlusion, aPLs can induce diffuse vascular wall proliferation, resulting in long-segmental stenosis (9). PV-aPL is a rare cause of vascular stenosis and may be an un(der)recognized form of extra-criteria manifestations of APS (10). Relatively little is known, however, about the angiographic features of PV-aPL involving the cerebral and cervical arteries (11).

The present study utilized magnetic resonance angiography (MRA) to analyze the angiographic features of PV-aPL involving the cerebral and cervical arteries in patients with aPLs who presented with neurologic symptoms. The purpose of this study was to investigate whether PV-aPL involving the cerebral and cervical arteries is an extra-criteria manifestation of APS in patients with aPLs.

## Methods

### Patients

Thirty patients with PV-aPLs who underwent brain and neck MRA for neurologic symptoms between January 2007 and October 2020 at Seoul National University Hospital were selected from electronic medical records. All patients satisfied the laboratory criteria of the revised Sapporo classification criteria for APS (12), and they had diffuse narrowing or diffuse luminal irregularity on a brain or neck MRA. After excluding 19 patients with atherosclerosis (*n* = 7), moyamoya disease (*n* = 11), genetic disorder (*n* = 1, underlying microcephalic osteo-dysplastic primordial dwarfism type II) and no significant vascular abnormality (*n* = 4), 11 patients were finally included in this study (Supplementary Figure S1). This study was approved by the Institutional Review Board of Seoul National University Hospital (IRB # 2004-081-1117), which waived the requirement for patient consent because of the retrospective nature of the study and no identifiable information was used. The study was conducted in accordance with the principles of the Declaration of Helsinki and Good Clinical Practice guidelines.

### Data Collection

Patients' electronic medical records were reviewed, and their demographic, clinical and laboratory data were recorded, with focus on neurologic manifestations and duration, comorbidities, cardiovascular risk factors (such as hypertension, diabetes, dyslipidemia and smoking status), treatment, and outcomes.

### Measurement of aPLs

aCLs (IgG and IgM) and anti-beta 2 glycoprotein I antibodies (aB2GPIs) (IgG and IgM) in serum were measured using standardized enzyme-linked immunosorbent assay. Lupus anticoagulant (LA) detected according to the guidelines of the International Society on Thrombosis and Haemostasis (13).

The presence of clinically significant aPLs was defined as anti-CL, anti-B2GP1 over 40 units, and/or positive LA on 2 or more occasions at least 12 weeks apart (12). A high-risk aPL profile was defined as positivity for at least two of the following three anti-bodies: LA, aCLs and aB2GPIs (14).

### Image Acquisition

Brain and neck MRAs were performed on 3.0 T MR scanners (Ingenia CX, Philips Healthcare, Best, the Netherlands; Discovery MR750w, GE Healthcare, Milwaukee, WI; or Magnetom Skyra, Siemens Healthineers, Erlangen, Germany) with a multi-channel head and neurovascular coils. Brain MRA was performed using a 3-dimensional multi-slab time-of-flight (TOF) MRA sequence for the arteries at the circle of Willis. Neck arteries were evaluated by TOF- or contrast-enhanced MRA.

Vascular wall and intraluminal changes were also evaluated in six subjects using high-resolution vessel wall MRI (VW-MRI). Proton density-weighted images and non-contrast and contrast-enhanced T1-weighted images were obtained with 3-dimensional fast spin-echo pulse sequences, followed by review of multiplanar reconstructed images. Detailed acquisition parameters are described in Supplementary Data S1.

### Image Analysis

MR imaging sequences were reviewed during the same session. Radiographic features included the involved cerebral or cervical arteries, the sites of arterial involvement, and the types of abnormalities including stenosis, aneurysm, and distal occlusion. Distal occlusion was defined as an occlusion of any segment of the anterior cerebral artery (ACA) or posterior cerebral artery (PCA), or an occlusion at or distal to the opercular segment of the middle cerebral artery (MCA)-M3 (15). Vascular wall thickening, intramural hematoma, and wall enhancement on VW-MRI were recorded, with wall thickening or enhancement categorized as concentric or eccentric. Proliferative vascuopathy was defined as a long-segmental stenosis due to vascular wall thickening without intraluminal occlusion or thrombus on MR images.

Two neuroradiologists (I.H. and C.H.S., with 9 and 31 years of experience in neuroradiology, respectively) reviewed all imaging results. Discrepancies between the investigators were resolved by consensus.

### Statistical Analysis

Continuous variables were reported as mean and standard deviation or as median and interquartile range (IQR), as appropriate. Categorical variables were reported in absolute numbers and percentages. All statistical analyses were performed with SPSS (IBM SPSS version 26.0, IBM Corp., USA).

## Results

### Patients Characteristics

Eleven patients, six men and five women, of median (IQR) age 42 years (34–61), had diffuse luminal narrowing affecting the cerebral and cervical arteries were included. Their main clinical symptoms, comorbidities, aPLs profiles, and other laboratory findings are summarized in Table 1. Six patients presented with acute neurologic symptoms, such as hemiplegia, syncope, diplopia, or memory impairment. The other five patients complained of chronic headache of duration 1 month to 5 years. Of these 11 patients, six, four and one had triple-, double- and single-positive aPLs profiles, respectively. Median aCLs (IgG) titer was 78.9 (28.2–134.0) units, and median aB2GPIs (IgG) titer was 227.2 (0.0–1012.1) units. D-dimer levels (normal ≤ 0.5 μg/mL) were elevated in only two patients, at concentrations of 1.39 and 0.54 μg/mL, respectively. C-reactive protein (CRP) concentrations were within normal range. Two patients had coexisting autoimmune conditions, including one with underlying systemic lupus erythematosus (SLE) and one with Graves' disease. Four patients were smokers and six had diabetes mellitus, dyslipidemia, and/or hypertension. No patients had significant coronary artery diseases, but one patient had valve vegetations (Patient 10). Two patients were positive for anti-nuclear antibody at concentrations ≥1:80.

**Table 1:** Clinical characteristics of patients with PV-aPL.

**Case no**.	**1**	**2**	**3**	**4**	**5**	**6**	**7**	**8**	**9**	**10**	**11**
Sex/Age	F/21	F/45	M/43	F/42	M/34	M/40	F/80	M/64	M/34	F/30	M/61
BMI, kg/m^2^	23.6	23.0	22.8	27.9	27.3	21.2	23.6	22.2	20.6	17.6	21.3
Clinical manifestation	Rt. arm motor weakness	Lt. side weakness	Headache	Headache	Headache	Headache	Lt. hemianopia	Syncope	Headache	Diplopia, dysarthria	Memory impairment
Symptom duration	2 weeks	6 hours	1 month	5 year	5 month	2 month	1 day	1 hour	2 years	2 days	2 hours
**Comorbidity**											
Hypertension	-	-	-	+	-	-	+	+	-	+	-
Diabetes mellitus	-	-	+	+	-	-	-	+	-	-	+
Dyslipidemia	-	-	-	+	-	-	-	-	-	-	-
Autoimmune disease	Graves' disease	-	-	-	-	-	-	-	-	SLE	-
Smoking status	Never	Never	Never	Never	Current	Never	Never	Current	Current	Never	Current
**aPLs profile**											
LA	+	N/A	+	+	+	+	+	N/A	+	+	+
aCL IgM, MPL unit	0	0	0	0	0	165.0	0	0	0	0	0
aCL IgG, GPL unit	832.9	57.8	184.2	123.4	71.9	16.1	108.0	28.2	21.1	134.0	78.9
aB2GPI IgM, unit	0	0	0	0	0	N/A	0	0	139.2	N/A	0
aB2GPI IgG, unit	4000.5	0	1487.6	410.0	227.2	N/A	536.6	118.0	0	N/A	0
ANA titer	1:40	0	1:40	1:40	N/A	0	1:160	0	1:40	1:80	N/A
ESR, mm/hr	7	16	3	4	15	12	49	16	56	23	8
CRP, mg/dL	0.03	0.03	0.04	0.12	0.12	0.41	0.56	0.05	0.13	0.02	0.18
D-dimer, ug/mL	0.54	0.08	0.17	0.50	0.15	N/A	0.13	N/A	1.39	0.35	N/A
**Treatment after Dx**											
Vitamin K antagonist	+	+	+	-	+	-	+	-	-	+	-
Aspirin	+	+	+	-	+	+	+	+	+	+	+
Clopidogrel	-	-	-	+	-	+	-	+	-	-	+
Cilostazole	-	-	-	-	-	-	-	-	+	-	-
Oral glucocorticoid	+	-	-	+	-	-	-	-	-	-	-
Hydroxychloroquine	+	-	+	-	+	+	-	+	-	-	+

*aB2GPI, anti-beta 2 glycoprotein I antibody; aCL, anticardiolipin antibody; ANA, anti-nuclear antibody; aPLs, antiphospholipid antibodies; BMI, body mass index; CRP, C-reactive protein; Dx, diagnosis; ESR, erythrocyte sedimentation rate; F, femal; LA, lupus anticoagulant; Lt, left; M, male; N/A, not available; PV, nonthrombotic proliferative vasculopathy; Rt, right; SLE, systemic lupus erythematosus*.

### Angiographic Characteristics of CNS Vasculopathy Associated With aPLs

**Figure 1:**
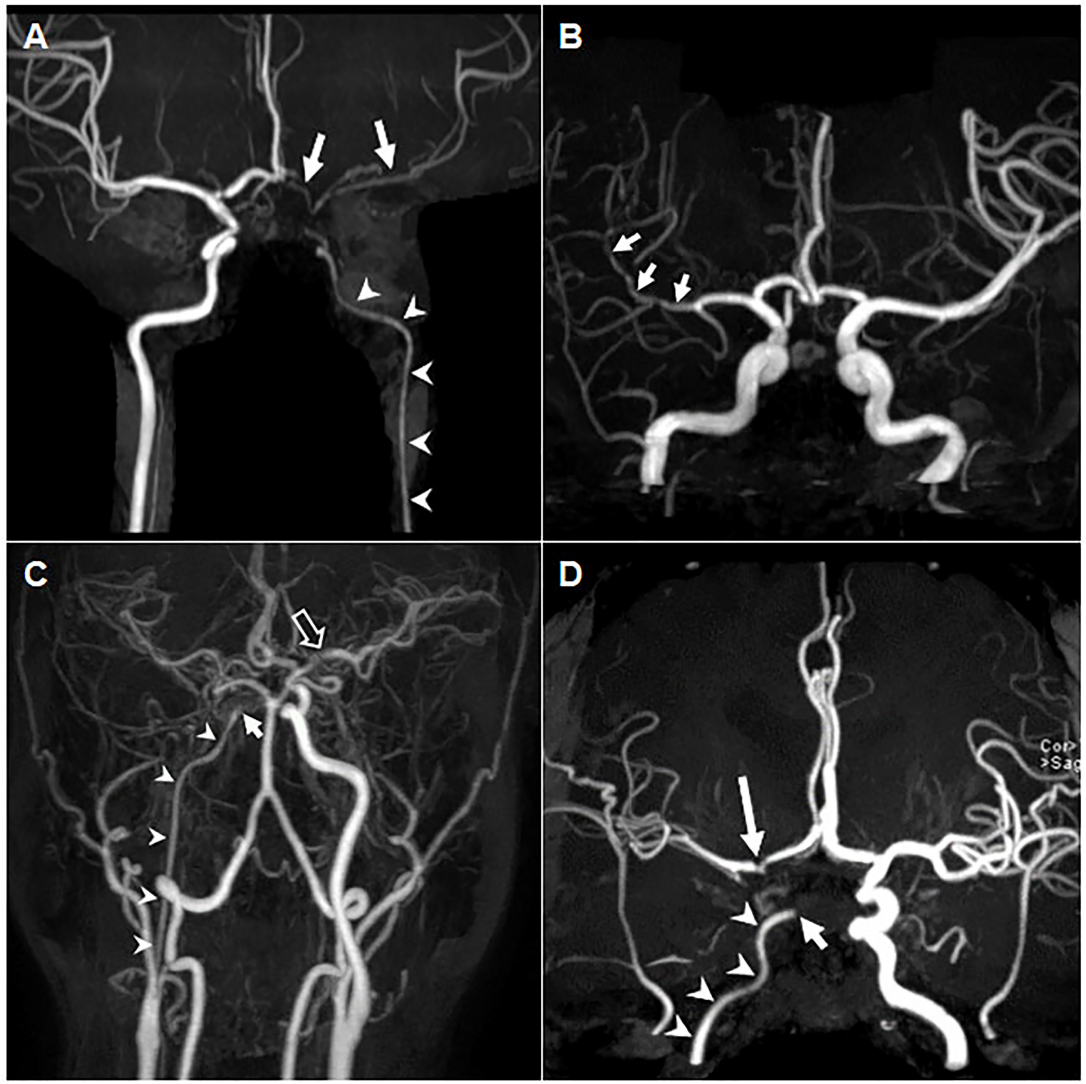
Long-segmental diffuse luminal stenosis of PV-aPL. **(A)** MRA of Patient 1, a 21-year-old woman with right arm weakness, showing a long-segmental diffuse stenosis of her left ICA (arrowhead), ACA and MCA (arrows). **(B)** Non-contrast MRA of Patient 2, a 45-year-old woman with left side weakness for 6 h, showing diffuse stenosis in the M1 distal to M2 segments of the right MCA (arrows). **(C)** Contrast-enhanced MRA of Patient 3, a 43-year-old man with a chronic headache for 1 month, showing diffuse stenosis of the right ICA (arrowheads) with distal occlusion (arrow), along with focal stenosis of the left MCA proximal to segment M1 (hollow arrow). **(D)** Non-contrast MRA of Patient 4, a 42-year-old woman with a chronic headache for 5 years and recurrent cerebral infarctions showed occlusion at a cavernous segment of the right ICA (short arrow) and long-segmental narrowing at the proximal ICA (arrowheads), with additional focal stenosis in the right ICA terminus (long arrow). aPLs, antiphospholipid antibodies; ICA, internal carotid artery; MCA, middle cerebral artery; MRA, magnetic resonance angiography; PV, non-thrombotic proliferative vasculopathy.

All 11 patients had diffuse luminal narrowing in major extra-cranial and cerebral arteries, five (45.5%) in the internal carotid artery (ICA); two (18.2%) in the MCA; two (18.2%) in the vertebral artery (VA); one (9.1%) in the basilar artery (BA); and one (9.1%) in the PCA (Table 2). MRA images of representative cases were shown in Figures 1A–D. One patient (Patient 1) had a long-segmental stenosis in the entire left ICA that extended into the left MCA (Figure 1A), whereas three patients (Patients 5, 7, and 8) had a relatively short-segmental narrowing (Supplementary Figure S2). Additional abnormalities in the same vascular territory as the diffuse narrowing, including focal stenosis in eight patients (72.7%); distal occlusion in three (27.3%); and an aneurysm in one (9.1%) were observed (Table 2).

**Table 2:** Details of radiographic characteristics of long-segmental stenosis.

**Case no**.	**Sites of the long-segmental luminal narrowing**					**Luminal abnormalities**			**Vascular wall abnormalities** ^†^			**Parenchymal change: infarction**
	**MCA**	**ACA**	**PCA**	**VBA**	**ICA**	**Focal stenosis**	Distal occlusion^*^	**Aneurysm**	**Thickening**	**Intramural hematoma**	**Wall enhancement**	
1	-	-	-	-	B +	+	-	-	C	-	C	Acute/subacute
2	R +	-	-	-	-	+	+	-	C	-	-	Acute
3	-	-	-	-	R +	-	+	-	C	-	-	Subacute
4	-	-	-	-	R +	+	-	-	C	-	C	Subacute
5	-	-	-	-	R +	+	-	-	E	-	E	Acute/subacute
6	-	-	-	B +	-	+	-	+	N/A	N/A	N/A	-
7	-	-	R +		-	+	-	-	N/A	N/A	N/A	Acute
8	-	-	-	-	L +	+	-	-	N/A	N/A	N/A	-
9	-	-	-	+	-	+	-	-	E	-	C	Acute
10	-	-	-	R +	-	-	-	-	N/A	N/A	N/A	-
11	R +	-	-	-	-	-	+	-	N/A	N/A	N/A	Acute

*ACA, anterior cerebral arteries; B, bilateral; C, concentric; E, eccentric; ICA, internal carotid artery; L, left; MCA, middle cerebral artery; N/A, not available; PCA, posterior cerebral artery; R, right; VBA, vertebral and basilar artery*.

* *Distal occlusion is defined as location of the occlusion in small arteries distal portion of the cerebral arteries*.

† *Vessel wall MRI finding*.

### Vascular Wall and Intraluminal Changes

**Figure 2:**
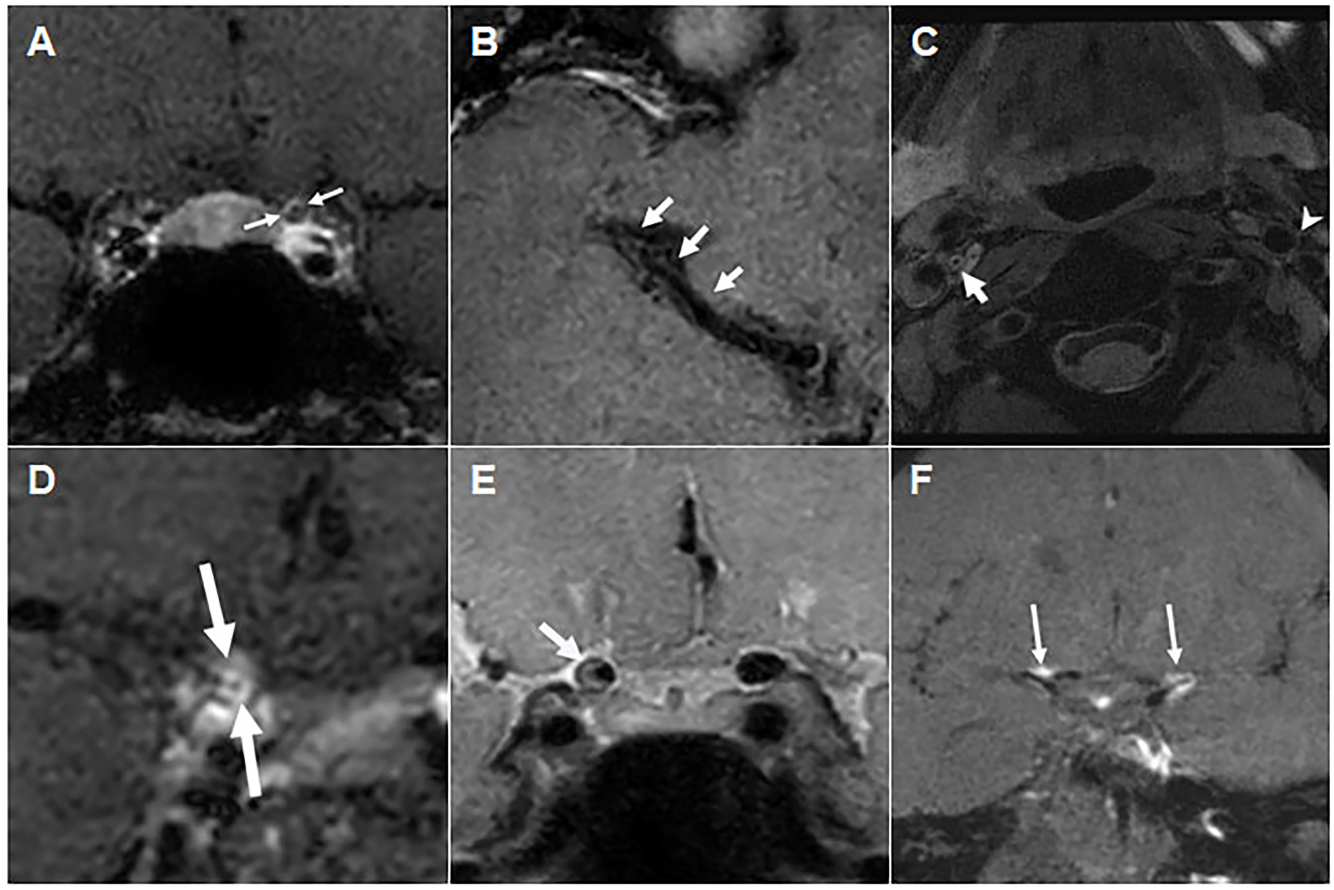
Vascular wall and intraluminal changes on high-resolution vascular wall MRI. **(A)** Post-contrast T1 weighted image of Patient 1, showing negative remodeling and diffuse concentric wall thickening (arrows) in the left distal ICA, along with mild contrast enhancement. **(B)** Contrast-enhanced T1 weighted image of Patient 2, showing a diffuse luminal narrowing of the right MCA M1 distal segment without contrast enhancement. **(C)** Proton density-weighted image of Patient 3, showing concentric intraluminal thickening of the right proximal ICA without definite occlusion and with no overt wall enhancement (post-contrast T1 weighted image not shown). **(D)** Contrast-enhanced T1 weighted image of Patient 4, showing concentric wall thickening and strong circumferential enhancement of the right ICA supraclinoid segment. **(E)** Proton density-weighted image of Patient 5, showing eccentric wall thickening with inner eccentric contrast enhancement (post-contrast T1 weighted image not shown) of the right distal ICA. **(F)** Contrast-enhanced T1 weighted image of Patient 9, showing multifocal vessel wall and subarachnoid nodular enhancement of bilateral ICA bifurcation and left ACA A1 segment (arrows). ACA, anterior cerebral arteries; aPLs, antiphospholipid antibodies; ICA, internal carotid artery; MCA, middle cerebral artery; PV, non-thrombotic proliferative vasculopathy; VW-MRI, vessel wall magnetic resonance imaging.

Six patients underwent high-resolution VW-MRI to assess vascular wall and intraluminal changes. VW-MRI showed concentric thickening of the vascular walls of the ICA and/or MCA in four patients, and mild eccentric thickening of the vascular walls of the ICA or BA in two patients (Figure 2). Three patients showed contrast enhancement of the vascular walls, including one with concentric (Figure 2E) and three with eccentric (Figures 2A,D,F) changes. None of these six patients had an intraluminal occlusion or thrombus, or an intramural hematoma (Table 2).

### Treatment

After diagnosis all patients received aspirin in combination with vitamin K antagonists or anti-platelet agents such as clopidogrel and cilostazol. Six patients received hydroxychloroquine and two were treated with systemic glucocorticoids (Table 1). Two patients underwent angioplasty, one (Patient 6) undergoing endovascular trapping of the dissecting aneurysm in the right distal VA, and the other (Patient 9) required a stent insertion for occlusion of the BA (Supplementary Figures S2B,E).

### Outcomes

**Figure 3:**
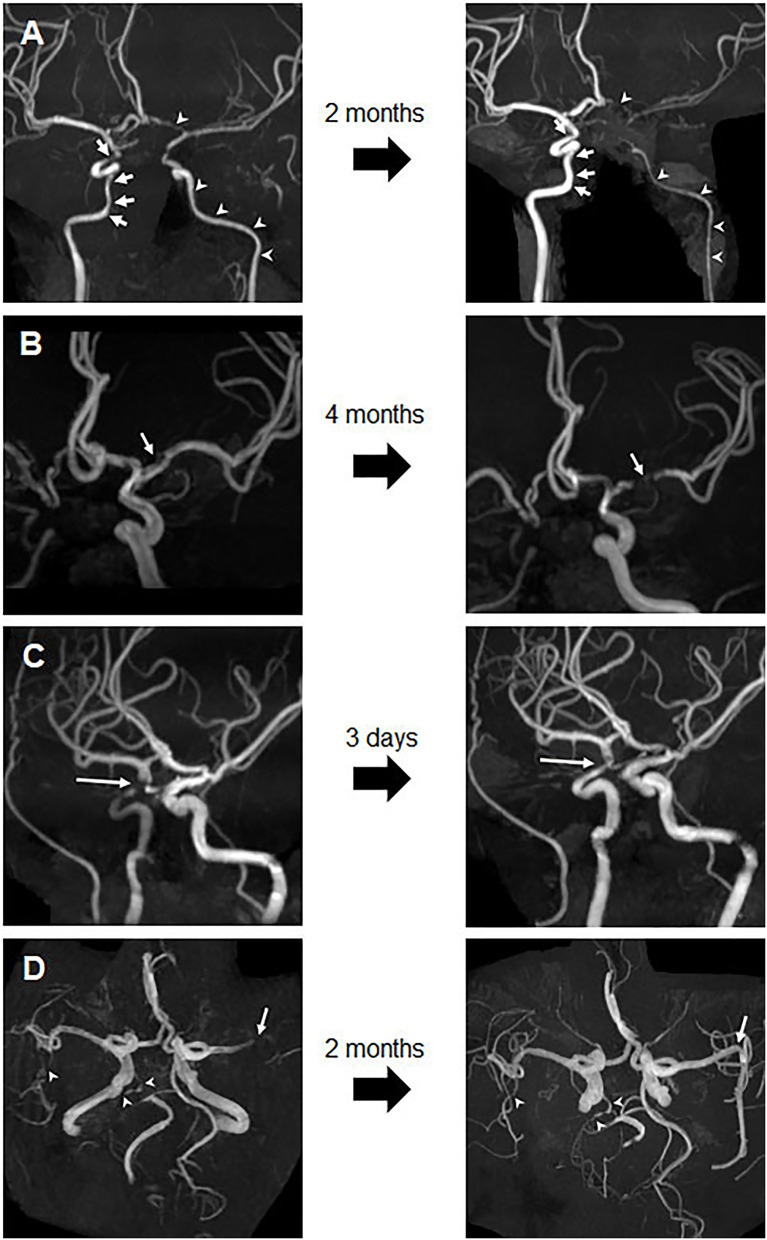
Changes in stenosis on follow-up images. **(A)** Follow-up image of Patient 1 after 2 months, showing progression of the long-segmental diffuse stenosis in the left ICA and ACA (arrowhead) and improvement of focal stenosis in the right ICA (arrow). **(B)** Follow-up image of Patient 3 after 4 months, showing progression of the left MCA stenosis (arrow). **(C)** Follow-up image of Patient 5 after 3 days, showing significant improvement in the stenosis of the right distal ICA. **(D)** Follow-up brain MRA image of Patient 7 after 2 months, showing complete resolution of occlusion and M2 stenosis of the left MCA M1 (arrow), but no significant interval change in right MCA M2 and right PCA near total occlusion (arrowheads). ACA, anterior cerebral arteries; ICA, internal carotid artery; MCA, middle cerebral artery; MRA, magnetic resonance angiography; PCA, posterior cerebral artery.

Eight patients underwent follow-up MRA after a median 110 days (IQR, 13.3–319.3 days) (Supplementary Table S1). Luminal changes in each vascular segment/territory varied between and within patients. In Patient 1, with extremely high levels of aCL IgG (832.9 GPL) and aB2GPI IgG (4000.5 GPL), long-segmental diffuse narrowing of the left ICA progressed, despite treatment with vitamin K antagonists, aspirin, hydroxychloroquine, and (high-dose) systemic glucocorticoids for possible vasculitis (Figure 3A). By contrast, focal stenosis in the right distal ICA showed improvement. In Patient 3 with extremely high levels of aCL IgG (184.2 GPL) and aB2GPI IgG (1487.6 GPL), the stenosis in the left MCA progressed (Figure 3B). By contrast, focal stenosis of the right distal ICA in Patient 5 (Figure 3C) and of the left MCA M1 in Patient 7 (Figure 3D) resolved completely after short-term anticoagulant treatment. Follow-up MRA imaging of the remaining three patients showed no significant interval changes.

## Discussion

This case series of 11 patients suggests that PV-aPL might be an un(der)recognized extra-criteria manifestation of APS affecting the cerebral and cervical arteries in relatively young patients with positive aPL who presented with neurologic symptoms. PV-aPL was characterized by relatively long-segmental stenosis and vascular wall thickening, but with no definite intraluminal thrombosis on MRA.

Neurologic manifestations of APS can be quite heterogeneous (16). The classic manifestations of APS include arterial, venous or small vessel thromboses provoked by thrombotic aPLs (12). TIA and stroke are the most common neurologic manifestations of APS due to *in-situ* thrombosis or thromboembolism (17). However, non-thrombotic neurologic symptoms of APS can manifest as headache, seizures, and movement disorders resulting from direct aPLs associated immune-mediated injury to the brain (18, 19). Here, all 11 patients with PV-aPL presented with TIA/stroke or headache, although it was unclear whether headache resulted from vasculopathy-related ischemia, was immune-mediated, or was even co-incidental. These patients with PV-aPL were relatively young, with a median age of 42 years. In contrast to stroke due to classic APS, which is three times as frequent in women as in men (20), PV-aPL did not show a female preference. Whereas, classical APS is often associated with other systemic autoimmune diseases, such as SLE, only one of the 11 patients with PV-aPL had SLE (21). A nation-wide population-based study estimated that the incidence of APS was 7.5 per 10^6^ person-year. Inasmuch as only 11 (2.6%) of the 425 initially screened patients with positive aPLs, neurologic symptoms, and MRA had PV-aPL, the true incidence of PV might be lower than that of classical APS.

A striking angiographic finding of PV-aPL is the extensive long-segmental stenosis of medium sized extra- and intracranial arteries (i.e., the carotid, basilary and proximal cerebral arteries). A similar angiographic finding of the aorta and its main branches in a patient with APS has been described as a “Takayasu” arteritis-like non-inflammatory vasculopathy (11). None of the patients met the criteria for primary systemic vasculitis including Takayasu arteritis or giant cell arteritis in this study, while Ree et al. (8) reported a prevalence of 6.3% of APS in primary systemic vasculitis (22). The current study shows that PV-aPL affects cerebral arteries in addition to pulmonary, visceral and peripheral arteries.

Many of these patients with PV-aPL had short-segmental (focal), abrupt stenosis, and distal occlusions, suggestive of atheromatous or thrombotic lesions (23). Patients with PV-aPL may be at higher risk of atherosclerosis, as the risk of developing atherosclerotic plaques in the carotid and femoral arteries patients is about 2.5-fold higher in patients with APS and SLE than in healthy controls (24–26). One patient in the present study (Patient 5) showed resolution of a short-segmental occlusion, with eccentric thickening and enhancement of the vascular walls on VW-MRI, following anticoagulation treatment, suggesting that classical APS can coexist with PV-aPL (27, 28). The association between atherosclerosis and the development of PV is unclear. Previous study reported an atherogenic role for IgG aCL using intima media thickness of carotid arteries screened by high-resolution sonography (29). Therefore, aPL might drive both PV and atherosclerosis. It is also possible traditional cardiovascular risk factors might contribute to PV pathogenesis as well. In this regard, optimal management of cardiovascular risks including lipid-lowering with statin and/or eicosapentaenoic acid might have positive short- or long-term effects on the PV-aPL (30).

In a previous study, two major types of intracranial arterial abnormalities of aPLs have been reported: stem occlusions of major arteries or branches, and multifocal sites of arterial narrowing and widening (11). However, several case studies have described moyamoya-like vasculopathy in patients with aPLs (31, 32). The long-segmental stenosis in our patients may belong to the latter category, but luminal angiography methods such as MRA have limited ability to visualize the underlying pathologic processes in these blood vessel. VW-MRI can directly image pathologic changes in vessel walls. In general, concentric wall thickening and enhancement observed on VW-MRI are suggestive of vasculitis (27). In contrast, intramural thrombosis is less likely to cause wall enhancement (33) but manifests as intraluminal enhancement (34), which was not observed in our patients. Furthermore, to our knowledge, no report published in English has described VW-MRI findings of aPLs. Therefore, the results of our study suggest that the characteristic long-segmental stenosis in our patients was not likely due to diffuse intraluminal thrombus formation but may be due to PV. The absence of an intraluminal thrombus, concentric vascular wall thickening on VW-MRI, normal d-dimer levels, and disease progression despite treatment with anticoagulants and anti-platelet agents suggested that long-segmental stenosis was not caused by “classical APS” with intraluminal thrombus. In addition, normal CRP levels and lack of response to corticosteroids also make inflammatory vasculopathy (i.e., vasculitis, such as Takayasu or polyarteritis nodosa) less likely (9, 35), although a mural contrast enhancement was observed in some patients.

Proliferation of the vascular wall may be driven by aPLs (36), which can activate endothelial cells that release proliferative cytokines. These cytokines promote the proliferation of cells in the intimal and media layers, leading to concentric stenosis similar to transplant vasculopathy and pulmonary arterial hypertension (37–39). It is not clear whether aPLs titers and/or certain aPLs profiles are associated with the extent and progression of PV-aPL. In a previous study by Djokovic et al. (40) the presence of aB2GPI IgG might be associated with more serious cerebrovascular events. Consistent with this, 2 patients with high aB2GPI IgG had progression of PV in follow up images in the current study. If aPLs play a direct role, then their reduction through plasmapheresis and/or depletion of B cells or plasma cells might improve long-term prognosis. As there is no official management guideline of the PV-aPL, further research is needed to define optimal treatment of PV-aPL.

This study has several limitations. First, it included only patients with neurologic symptoms, aPLs and “diffuse luminal narrowing” on MRA. The incidence of PV-aPL in asymptomatic aPLs patients remains unknown. In addition, PV-aPL might also manifest as a focal stenosis and those might be missed in this case series. Second, in the absence of the histology, it is not possible to exclude vasculitis based on MRI findings (although there was no clinical and laboratory evidence suggestive of systemic vasculitis in this patient cohort). Since the characteristic MRI findings of proliferative vasculopathy remains to be defined, it is not clear whether contrast enhancement seen on MRI is specific for proliferative vasculopathy and/or vasculitis. Further research is required to define the characteristic MRI findings of proliferative vasculopathy. Third, we excluded the patients with moyamoya disease or moyamoya syndrome since it can potentially mimic or be even a form of proliferative vasculopathy associated with aPL. One might speculate that PV-aPL affecting carotid stenosis could progress and involve small cerebral vessels over time, mimicking moyamoya appearance. Moreover, although systemic vasculitis was not an exclusion criterion, none of the 11 included patients had primary systemic vasculitis. Since patients with systemic vasculitis often have aPL, further research is needed to evaluate the impact of aPL associated with systemic vasculitis on proliferative vasculopathy. Finally, this case series with a small number of patients needs to be validated in other populations. Further studies are needed to assess the epidemiology, natural course and optimal treatment of PV-aPL.

## Conclusions

This study suggests that PV**-**aPL is a distinct extra-criteria manifestation of APS that can manifest as a long-segmental diffuse stenosis of the cerebral and cervical arteries. PV**-**aPL should be included in the differential diagnosis of young patients with neurologic symptoms and aPLs.

## Data Availability Statement

The original contributions presented in the study are included in the article/Supplementary Material, further inquiries can be directed to the corresponding author.

## Ethics Statement

The studies involving human participants were reviewed and approved by Institutional Review Board of Seoul National University Hospital. Written informed consent for participation was not required for this study in accordance with the national legislation and the institutional requirements.

## Author Contributions

JKP had full access to all of the data in the study and takes responsibility for the integrity of the data and the accuracy of the data analysis. JY, IH, and JKP contributed to the study concept and design. JY, IH, CHS, EEL, STL, EBL, and JKP contributed to the acquisition, analysis, interpretation of the data, and contributed to the critical revision of the manuscript for important intellectual content. JY, IH, and JKP drafted the manuscript and contributed to the data interpretation. All authors read and approved the final manuscript.

## Funding

This work was supported by the National Research Foundation of Korea (NRF) grant funded by the Ministry of Science, ICT and Future Planning, Republic of Korea (grant number: 2019R1G1A1100477).

## Conflict of Interest

The authors declare that the research was conducted in the absence of any commercial or financial relationships that could be construed as a potential conflict of interest.

## Publisher's Note

All claims expressed in this article are solely those of the authors and do not necessarily represent those of their affiliated organizations, or those of the publisher, the editors and the reviewers. Any product that may be evaluated in this article, or claim that may be made by its manufacturer, is not guaranteed or endorsed by the publisher.
