# Supplementary material for: Proliferative Vasculopathy Associated With Antiphospholipid Antibodies in Patients With Neurological Symptoms
Source: Front Med (Lausanne). 2022 Jun 20;9:913203. doi: 10.3389/fmed.2022.913203 (PMC9252304; doi:10.3389/fmed.2022.913203)
Supplement: Supplementary file 1 [file Data_Sheet_1.PDF]

Table of Contents

Supplementary Method S1

Magnetic resonance angiography (MRA) acquisition **Page 2**

Vessel wall magnetic resonance imaging (MRI) acquisition **Page 3**

Supplementary Figure S1. **Page 4**

Supplementary Figure S2. **Page 5**

Supplementary Table S1. **Page 6**

Supplementary Method S1

Magnetic resonance angiography (MRA) acquisition.

Brain and neck MRAs were performed on 3.0 T MR scanners (Ingenia CX, Philips Healthcare, Best, the Netherlands; Discovery MR750w, GE Healthcare, Milwaukee, WI; or Magnetom Skyra, Siemens Healthineers, Erlangen, Germany) with a multi-channel head and neurovascular coil. The imaging parameters were optimized for each scanner according to the manufacturer's recommendation for routine clinical imaging. The noncontrast brain or neck MRA was obtained using a 3-dimensional (3D) multi-slab time-of-flight (TOF) MRA sequence with ramped flip angle. Brain MRA imaged the arteries at the circle of Willis, while neck MRA included the carotid and vertebral arteries from the aortic arch to the skull base. The acquisition parameters are as follows: repetition time (TR), 20-23 ms; echo time (TE), 3.4-3.7 ms, flip angle (FA), 18-25°; acquisition matrix, 448-536 × 256-378 (brain) or 320-384 × 187-192 (neck); field-of-view (FOV), 210-240 × 210-240 mm^2 (brain) or 199-300 × 149-300 mm^2 (neck); slice thickness, 1.2-1.4 mm (brain) or 1.5-2 mm (neck); slice interval, 0.6-0.7 mm (brain) or 1-1.5 mm (neck); number of excitations (NEX), 1. A superior saturation slab was applied to suppress the venous flow signal. Fat suppression was used in brain MRA. The contrast-enhanced brain and neck MRA was obtained using a 3D fast-spoiled gradient-refocused echo sequence after intravenous administration of 0.1 mmol/kg of gadobutrol (Gadovist; Bayer Pharma AG, Berlin, Germany). The imaging parameters as follows: TR 3.4-3.8 ms; TE 1.3-1.5 ms; FA, 20-27°; acquisition matrix, 320-576 × 256-503; FOV 320-350 × 320-350 mm^2; NEX, 1; imaging plane, coronal. The maximum intensity projection images were evaluated for imaging review.

Vessel wall magnetic resonance imaging (MRI) acquisition

High-resolution vessel wall MRI was performed on 3.0 T MR scanners (Ingenia CX or

Discovery MR750w) with a multi-channel head coil. Our vessel wall MRI protocol included 3D proton density-weighted imaging (PDWI), pre and post-contrast T1-weighted imaging (T1WI), and routine brain MRA sequence. The 3D PDWI was obtained using the 3D fast spin-echo or Cube (GE Healthcare) pulse sequences with the following parameters: TR, 1800-2000 ms; TE, 36.8-40.2 ms; FA, 90°; echo train length (ETL), 63-76; acquisition matrix, 300-360 × 300-360; FOV, 180 × 180 mm^2; slice thickness, 0.5-0.8 mm; slice interval, 0.25-0.4 mm; NEX, 1; imaging plane, coronal. The pre and post-contrast 3D T1WI were obtained before and after the intravenous administration of 0.1 mmol/kg of gadobutrol, using the 3D fast spin-echo or Cube (GE Healthcare) pulse sequences with the following parameters: TR, 591-650 ms; TE, 29.3-29.6 ms; FA, 90°; echo train length (ETL), 28-46; acquisition matrix, 260-292 × 260-292; FOV, 180-199 × 180-199 mm^2; slice thickness, 0.6-0.8 mm; slice interval, 0.3-0.4 mm; NEX, 1-2; imaging plane, coronal. The obtained images included the upper cervical arteries and circle of Willis arteries**.** Motion-sensitized driven-equilibrium preparations were applied to obtain black-blood imaging in all pulse sequences. Three orthogonal multiplanar reconstructed images were routinely generated, and longitudinal and perpendicular plane to the arterial segment of interest were also produced by a neuroradiologist (I.H.).

Supplementary Figure S1. Patient flow diagram.

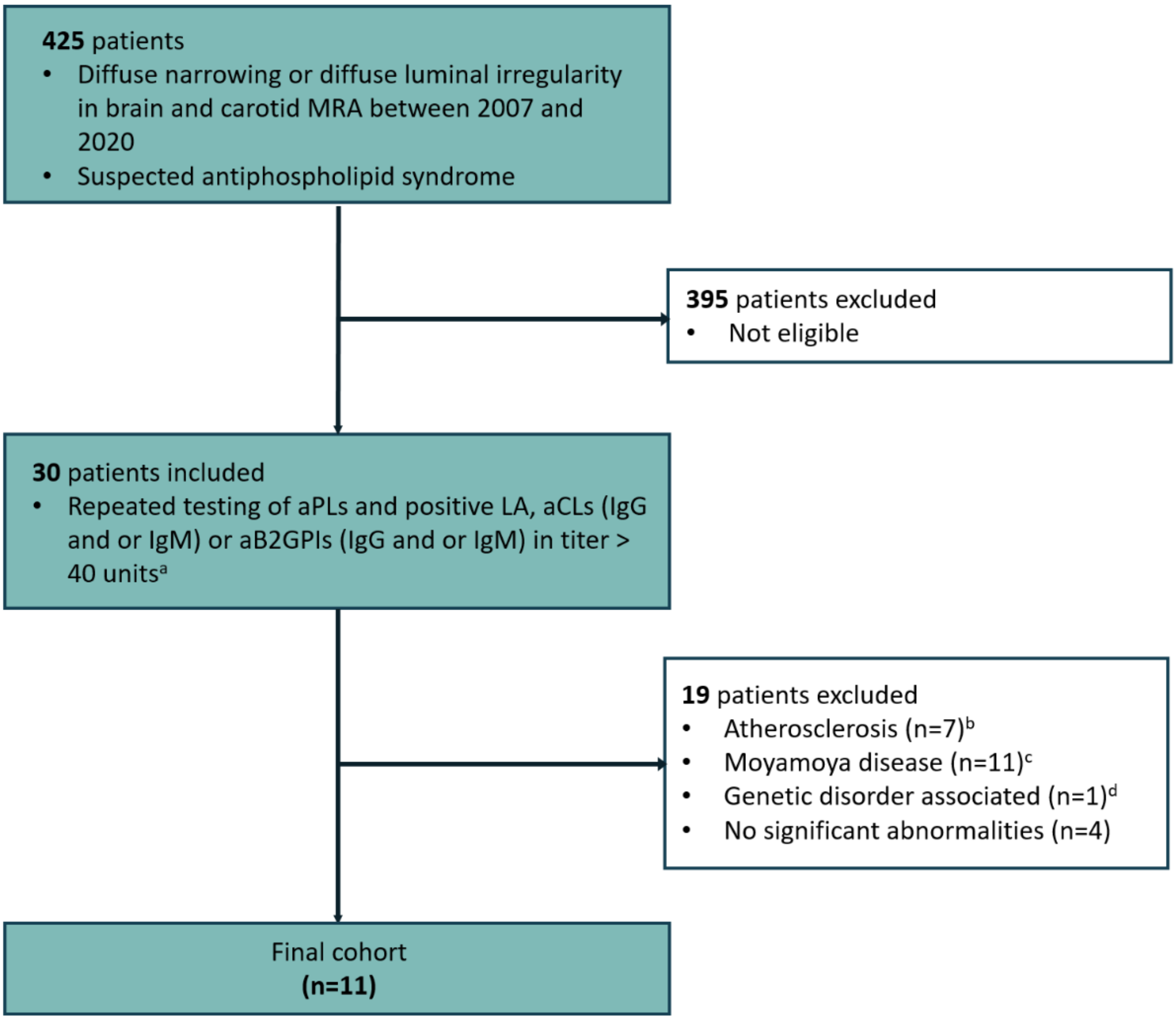

[a]LA, aCL IgG and/or IgM, and aB2GPI IgG and/or IgM in serum or plasma positive on ≥2 occasions at least 12 weeks apart
[b]Atherosclerosis was defined by the short-segmental stenosis, mural calcification and/or plaque at branch points in cerebral or cervical arteries on MRI images.
[c]Moyamoya disease was diagnosed according to the radiographic criteria [Neurol Med Chir (Tokyo). 2012;52(5):245-66].
[d]The patient with underlying microcephalic osteodysplastic primordial dwarfism type II was excluded.

aCLs, anticardiolipin antibodies; aB2GPIs, anti-beta 2 glycoprotein I antibodies; LA, lupus anticoagulant

Supplementary Figure S2. Brain or neck MRA images in patients with NTPV aPL s.

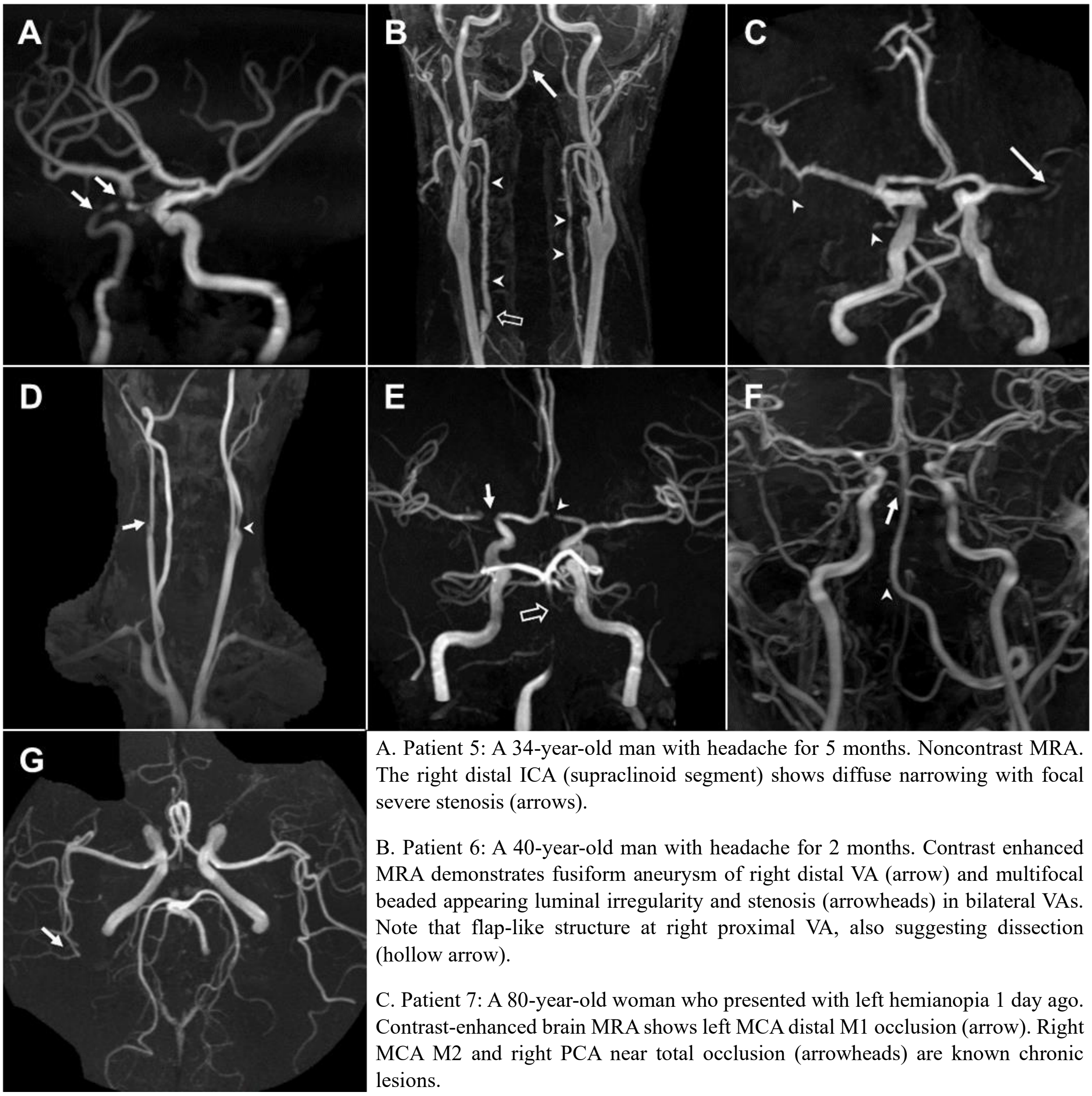

A. Patient 5: A 34-year-old man with headache for 5 months. Noncontrast MRA. The right distal ICA (supraclinoid segment) shows diffuse narrowing with focal severe stenosis (arrows).

B. Patient 6: A 40-year-old man with headache for 2 months. Contrast enhanced MRA demonstrates fusiform aneurysm of right distal VA (arrow) and multifocal beaded appearing luminal irregularity and stenosis (arrowheads) in bilateral VAs. Note that flap-like structure at right proximal VA, also suggesting dissection (hollow arrow).

C. Patient 7: A 80-year-old woman who presented with left hemianopia 1 day ago. Contrast-enhanced brain MRA shows left MCA distal M1 occlusion (arrow). Right MCA M2 and right PCA near total occlusion (arrowheads) are known chronic lesions.

D. Patient 8: A 64-year-old man with a syncope. Noncontrast neck MRA revealed total occlusion of right proximal ICA (arrowhead) and severe stenosis at left proximal ICA (arrow).

E. Patient 9: A 34-year-old male who had suffered headache for 2 years. On noncontrast MRA, there is severe stenosis lesion at right MCA M1 proximal segment (arrow) and left ACA A1-2 segments (arrowhead). The BA is poorly visualized due to previously deployed stent (hollow arrow).

F. Patient 10: A 30-year-old female who presented with diplopia and dysarthria 2 days ago and revealed valve vegetation. Contrast-enhanced MRA reveals chronic occlusions of right SCA (arrow) and AICA (arrowhead).

G. Patient 11: A 61-year-old male who reported the onset of memory impairment 2 hours prior to examination. Noncontrast brain MRA shows right M2 inferior division distal branch (angular artery) occlusion (arrow).

ACA, anterior cerebral arteries; AICA, anterior inferior cerebellar artery; aPLs, antiphospholipid antibodies; BA, basilar artery; ICA, internal carotid artery; MCA, middle cerebral artery; MRA, magnetic resonance angiography; NTPV, nonthrombotic proliferative vasculopathy; PCA, posterior cerebral artery; SCA, superior cerebellar artery; VA, vertebral artery

Supplementary Table S1. Summary of interval changes in follow up image study and maintenance treatment.

Case no.	Time interval between MRA scans (days)	Interval changes of MRA findings
1	179	Progression of diffuse stenosis in left ICA and focal stenosis in left ACA and MCA<br>Improvement of focal stenosis in right distal ICA
2	143	No interval change of diffuse stenosis in right MCA
3	1154	Progression of focal stenosis in left MCA<br>No interval change of diffuse stenosis in right ICA
4	1	No interval change of diffuse stenosis in right ICA
5	3	Rapid improvement of stenotic lesion in right ICA
6	366	State of trapping of dissecting aneurysm and no significant interval change of diffuse stenosis in right distal VA
7	77	Complete resolution of left MCA occlusion<br>No interval change of diffuse stenosis in right PCA
8	N/A	
9	44	Patent stent placement in BA<br>Interval development of severe stenosis at right MCA<br>No interval change of severe stenosis in left ACA.
10	N/A	
11	N/A	

ACA, anterior cerebral arteries; BA, basilar artery; ICA, internal carotid artery; MCA, middle cerebral artery; MRA, magnetic resonance angiography; N/A, not available; PCA, posterior cerebral artery; VA, vertebral artery
